# Microstructural alterations in brain tissue of ME/CFS and long COVID using diffusion tensor imaging and diffusion kurtosis imaging

**DOI:** 10.3389/fmed.2026.1824498

**Published:** 2026-07-17

**Authors:** Tanoj Bahadur Singh, Sonya Marshall-Gradisnik, Leighton Barnden, Natalie Eaton-Fitch, Tuong Hieu Huynh, Maira Inderyas, Kiran Thapaliya

**Affiliations:** National Center for Neuroimmunology and Emerging Diseases, Griffith University, Gold Coast, QLD, Australia

**Keywords:** Myalgic Encephalomyelitis/Chronic Fatigue Syndrome, ME/CFS, diffusion tensor imaging, DTI, diffusion kurtosis imaging, DKI, long COVID, MRI

## Abstract

**Introduction:**

Myalgic Encephalomyelitis/Chronic Fatigue Syndrome (ME/CFS) and long COVID have overlapping symptoms, such as profound fatigue, cognitive impairment, post-exertional malaise, pain and sleep disturbances that are debilitating and reduce quality of life. While Magnetic Resonance Imaging (MRI) techniques, specifically Diffusion Tensor Imaging (DTI) and Diffusion Kurtosis Imaging (DKI), have been used to investigate brain tissue microstructure in ME/CFS or long COVID, no study has yet combined these modalities to directly compare tissue microstructural differences between people living with ME/CFS and long COVID.

**Methods:**

We recruited 37 ME/CFS participants (Age: 43.56 ± 12.5), 19 long COVID participants (Age: 47.92 ± 13.3), and 27 healthy controls (Age: 37.9 ± 10.4). Data were acquired using a 3 Tesla (3T) Prisma MRI scanner. DTI and DKI metrics were determined using MRtrix v3.0.7 and Designer V2.0 software, respectively. Voxel-based statistical analysis of cohort differences was performed using the Statistical Parametric Mapping (SPM12) toolbox in MATLAB. Correlation analysis was performed between DTI, DKI metrics and clinical measures such as duration of illness, fatigue severity, SF36 domains and WHODAS domains.

**Results:**

Compared with healthy controls, individuals with ME/CFS showed microstructural alterations in the cingulum, supplementary motor areas, and parts of the corpus callosum (all *p* < 0.05). Long COVID participants demonstrated microstructural alterations in regions including the fusiform and precentral gyrus and in major white matter tracks (all *p* < 0.05). Direct comparisons between ME/CFS and long COVID revealed difference in the left corona radiata (*p* = 0.001).

**Conclusion:**

This study identifies distinct tissue microstructural alterations in ME/CFS and long COVID and offers a vital insight into the neuropathological basis of shared symptoms in both conditions.

## 1 Introduction

People with Myalgic Encephalomyelitis/Chronic Fatigue Syndrome (ME/CFS) and long COVID experience a range of shared symptoms, including severe fatigue, cognitive difficulties, post-exertional malaise, pain, and disrupted sleep, that are highly disabling and cause a significant decrease in quality of life (1, 2). Currently, there is no universal diagnostic test for ME/CFS or long COVID (3). The diagnosis of ME/CFS is based on standardized case definitions such as the Canadian Consensus Criteria (CCC) (4) and International Consensus Criteria (ICC) (5). Similarly, the diagnosis of long COVID uses the World Health Organization (WHO) case definition (6), a detailed medical history, and the patient's physical examination to rule out other conditions (7, 8).

Under the WHO International Classification of Diseases, ME/CFS is classified as a disease of the nervous system (9). Long COVID also presents with various neurological symptoms (10). Cognitive impairment is a prominent central nervous system (CNS) related symptom in both conditions, often presenting as difficulties with memory, concentration, or “brain fog” (11, 12). Other CNS-related symptoms such as fatigue, sleep disturbances, headache and pain are also present in these conditions (11). Therefore, neuroimaging techniques such as MRI have been used to study brain dysfunction in ME/CFS and long COVID (13, 38). Diffusion weighted MRI (DWI), which measures the diffusion of water molecules, is a non-invasive technique to study brain microstructure, such as axons, glial cells and myelin (14).

Diffusion Tensor Imaging (DTI) is a specific type of modeling of the DWI datasets, which quantifies tissue water diffusion and enables the indirect measurement of the degree of diffusion anisotropy and its structural orientation (15). DTI is based on the Brownian motion of water molecule diffusion and is anisotropic along the axonal pathway, as perpendicular diffusion to the fiber is hindered by myelin sheaths, axonal cellular membranes and the neurofibrils (16, 17). This helps to estimate the axonal organization in the brain and provides image contrast based on structural orientation thereby indirectly assessing neuroanatomy structure at a microscopic level (17). The diffusion tensor has three perpendicular eigenvectors and three positive eigenvalues (18). Fractional Anisotropy (FA), the most widely used scalar, is basically a normalized variance of the eigenvalues (18). Mean Diffusion (MD) is the mean diffusion of each direction, which is the average of three eigenvalues (19). Axial Diffusion (AD) describes the diffusion rate along the primary axis, which is the largest eigenvalue of diffusion, while Radial Diffusion (RD) reflects the average diffusivity along the two minor axes (19).

Diffusion Kurtosis Imaging (DKI), an extension of DTI, enables the estimation of the diffusion kurtosis tensor to characterize additional non-Gaussian diffusion properties within complex biological tissues by estimating the excess kurtosis of the displacement distribution (20, 21). DKI has the ability to capture non-Gaussian diffusion and is more sensitive to microstructural complexity, although it requires a multishell acquisition protocol and has comparatively lower spatial resolution (22, 23). Similar to DTI, DKI possesses corresponding scalars for measuring diffusion kurtosis, including kurtosis fractional anisotropy (KFA), mean kurtosis (MK), axial kurtosis (AK) and radial kurtosis (RK) (24). KFA summarizes the directional variation in the degree of non-Gaussian diffusion (25). Similarly, MK quantifies the magnitude of diffusion kurtosis along the three spatial axes (26). Likewise, AK quantifies the magnitude of diffusion kurtosis along the main axis, and RK quantifies the magnitude of diffusion kurtosis perpendicular to the main axis (26).

DTI and DKI parameters have been widely used to investigate microstructural changes in the brain across various neurological conditions (27). People living with ME/CFS and long COVID also experience overlapping neurological symptoms, including brain fog, fatigue, cognitive impairment, and sleep disturbances (11, 12). Given these shared clinical features, examining both the similarities and differences in brain tissue microstructural alterations is important to improve our understanding whether these two conditions are similar or distinct. Thapaliya et al. (28) has demonstrated the microstructural changes in the brainstem region using DTI where AD and MD were significantly decreased whereas RD was increased in people with ME/CFS compared to healthy controls (28). Yu et al. reported ME/CFS individuals with post-infectious onset showed increased AD, whereas those with gradual onset exhibited reduced AD (29). Zeineh et al. (30) reported increased FA in the right anterior arcuate, while Kimura et al. (31) observed significantly lower FA in the genu of the corpus callosum and in the anterior limb of the internal capsule of ME/CFS compared to healthy controls. Furthermore, in people living with long COVID, Liang et al. (32) reported increases in FA in several white matter regions and in MD in the left amygdala. Studies have also reported significantly decreased FA, MD, RD and AD in long COVID compared to healthy controls (33, 34). Additionally, Thapaliya et al. demonstrated significantly higher FA value in the right superior longitudinal fasciculus in long COVID compared to COVID-recovered healthy controls and significantly lower AD and MD in the left caudate in COVID-recovered healthy control compared to non-COVID-healthy controls (6). Using the DKI method, Kimura et al. (31) found a significant decreases in the DKI metric MK in the right frontal area, anterior cingulate cortex, superior longitudinal fasciculus and left parietal areas. Yuan et al. (35) also found reduced MK and RK in the right inferior fronto-occipital fasciculus in recovered COVID-19 patients. Overall, inconsistencies in ME/CFS DTI findings were driven by varying diagnostic criteria, heterogeneous symptoms, sample size, data processing pipelines and the scarcity of DKI studies in ME/CFS and long COVID, limits the robustness of the current conclusions.

Given the equivocal nature of current findings, further investigation of tissue microstructural alterations in ME/CFS and long COVID is critical. Furthermore, no study has yet assessed DTI and DKI methods to directly compare tissue microstructural alterations between ME/CFS and long COVID. Therefore, this study aims to compare tissue microstructural alterations between ME/CFS, long COVID, and healthy controls using DTI and DKI metrics. Additionally, this study will explore the correlation between DTI and DKI metrics and cognitive measures in both ME/CFS and long COVID.

## 2 Materials and methods

### 2.1 Participant recruitment

The study was approved by the Griffith University Human Research Ethics Committee (Ref: 2022/666) and conducted in accordance with the principles outlined in the Declaration of Helsinki. Written informed consent was obtained from all participants prior to inclusion in the study.

This cross-sectional study was carried out in the National Center for Neuroimmunology and Emerging Diseases (NCNED), Gold Coast, Australia. Participants were recruited via the NCNED research registry database, as previously described in Thapaliya et al. (34). 37 ME/CFS participants were recruited after meeting the Canadian Consensus Criteria (CCC) (4) or International Consensus Criteria (ICC) (5) and had received a formal diagnosis of ME/CFS by a physician. 19 long COVID participants were recruited after meeting the WHO working case definition (6). 27 healthy controls were recruited if they reported no chronic health conditions, underlying illness and had no prior COVID-19 infection. Medical histories for diseased and control participants were reviewed to identify comorbid symptoms or exclusionary diagnoses, including mental illness, malignancies, autoimmune, neurological, or cardiovascular diseases. Female participants were excluded if they were pregnant and/or breastfeeding. For ME/CFS and long COVID, exclusionary mental illness applies only if they were present before the illness was diagnosed. Furthermore, pregnancy and breastfeeding were excluded due to safety issue of fetus during MRI scans. Additionally, pregnancy and breastfeeding are associated with substantial hormonal and metabolic changes that can influence brain microstructure, introducing potential confounding effects in diffusion-based measurements (36, 37).

### 2.2 Clinical measures

The National Center for Neuroimmunology and Emerging Diseases (NCNED) in conjunction with the Centers for Disease Control and Prevention (CDC), has developed the research registry questionnaire and distributed it online through Lime Survey and Redcap, as reported in a previous publication (38). The CDC 2005 Symptom Inventory is a self-report questionnaire designed by the CDC regarding physical symptoms that a ME/CFS patient may have experienced during the past month (39). One of the questionnaires captured fatigue severity from the ICC (5) and CCC (4) criteria. The inventory also asks the participant to rate his/her experience in the past month regarding unusual fatigue (39). Fatigue severity was self-reported using the CDC's Symptom Inventory, which scores symptoms on a five-level Likert scale: 1 = very mild, 2 = mild, 3 = moderate, 4 = severe, and 5 = very severe (39). Fatigue was considered present if it was reported as being at least very mild within the month prior to completing the questionnaire (40). Quality of life was measured using the 36-item Short-Form Health Survey, version 2 (SF-36v2) (98). Domains of SF-36v2 includes general health, physical functioning, role physical, role emotional, pain, mental health, vitality and social functioning. Scores for each domain fall between 0 and 100%, reflecting the individual's overall level of quality of life, with higher percentages indicating better perceived wellbeing (40). Functional capacity was evaluated using the World Health Organization Disability Assessment Schedule 2.0 (WHODAS 2.0), a standardized tool for assessing everyday functioning (97). Domains of WHODAS 2.0 includes cognitive, mobility, self-care, interpersonal-relation, life activity and society. Each domain produces a score from 0 to 100%, reflecting the extent of functional difficulty or disability in that area. Higher percentages indicate greater impairment, with 0% representing no limitation and 100% indicating very severe difficulty (40). Duration of illness for ME/CFS and long COVID following symptom onset was determined based on participant self-reports and formal diagnoses made by medical professionals. Table 1 shows demographic and clinical characteristics of ME/CFS, long COVID, and healthy controls.

**Table 1:** Demographic and clinical characteristics.

Variables	ME/CFS (*n* = 37)	Long COVID (*n* = 19)	Healthy controls (*n* = 27)	*P*-Value	Missing data	
					ME/CFS	Long COVID
**Age (years)**	43.56 ± 12.5	47.92 ± 13.3	37.9 ± 10.4	0.20^a^ 0.021^b^ 0.618^c^	N/A	N/A
**Sex (F/M)**	30/7	14/5	19/8	N/A	N/A	N/A
**Duration of illness (years)**	14.53 ± 12.3	0.78 ± 0.69	N/A	<0.001	6	1
**Fatigue severity**	3.73 ± 0.82	3.39 ± 0.5	N/A	0.176	7	1
**SF36 general health**	31.6 ± 13.7	55.9 ± 22.5	80.9 ± 10.2	<0.001^a^ <0.001^b^ 0.002^c^	10	6
**SF36 physical functioning**	37.2 ± 27.8	75.7 ± 7.3	90.9 ± 24.9	<0.001^a^ 0.028^b^ 0.017^c^	11	1
**SF36 role physical**	13.9 ± 17.8	43.7 ± 20.4	97.6 ± 6.4	<0.001^a^ <0.001^b^ 0.008^c^	11	5
**SF36 role emotional**	57.1 ± 38.5	71.4 ± 24.9	95.8 ± 7.4	<0.001^a^ 0.002^b^ 0.143^c^	11	3
**SF36 pain**	44.0 ± 24.9	61.0 ± 29.2	88.1 ± 15.8	<0.001^a^ <0.001^b^ 0.259^c^	9	4
**SF36 mental health**	58.4 ± 20.3	68.5 ± 17.9	85 ± 11.6	<0.001^a^ 0.002^b^ 0.09^c^	10	3
**SF36 vitality**	11.6 ± 10.9	34.8 ± 23.6	76.5 ± 12.8	<0.001^a^ <0.001^b^ 0.005^c^	13	6
**SF36 social functioning**	22.1 ± 19.2	55.3 ± 20.2	94.5 ± 9.0	<0.001^a^ <0.001^b^ 0.007^c^	11	6
**WHODAS cognitive impairment**	49.6 ± 14.0	36.2 ± 22.7	3.0 ± 5.5	<0.001^a^ <0.001^b^ 0.004^c^	9	3
**WHODAS mobility**	48.5 ± 21.0	31.1 ± 20.5	0.33 ± 1.2	<0.001^a^ <0.001^b^ 0.158^c^	9	6
**WHODAS selfcare**	28.1 ± 25.5	13.2 ± 19.3	0.00 ± 0.00	<0.001^a^ 0.01^b^ 0.482^c^	8	2
**WHODAS interpersonal**	40.8 ± 26.7	35.3 ± 32.6	1.6 ± 3.7	<0.001^a^ 0.005^b^ 0.079^c^	9	7
**WHODAS life activity**	69.5 ± 21.0	42.3 ± 27.9	1.6 ± 4.3	<0.001^a^ <0.001^b^ 0.007^c^	9	4
**WHODAS society**	63.8 ± 15.8	42.5 ± 18.6	1.6 ± 3.5	<0.001^a^ <0.001^b^ <0.001^c^	9	3

Values are given as mean ± standard deviation for ME/CFS, long COVID and Healthy controls. Multiple comparison correction was performed using the Bonferroni method. For the three P-values superscript ‘a' represents ME/CFS vs healthy controls, ‘b' represents long COVID vs healthy controls and ‘c' represents ME/CFS vs. long COVID. M: Male, F: Female, n number of subjects, ME/CFS: Myalgic Encephalomyelitis/Chronic Fatigue Syndrome.

### 2.3 Imaging parameters

The diffusion data were acquired using a 3Tesla Prisma (Siemens Scanner) MRI scanner with a 64-channel head-neck coil. DTI data were acquired using 2-shell acquisition protocols: 30 directions at *b* = 1,000 s/mm^2^ and 66 directions at *b* = 2,500 s/mm^2^, along with nine *b* = 0 scans. Other settings were repetition time/echo time = 4,100/75 ms, field of view (FOV) = 244 × 244, flip angle = 90°, matrix = 122 × 122, voxel dimension of 2.0 mm^3^ and 66 slices, phase encoding direction: anterior to posterior (AP).

### 2.4 Data processing

In this study, for DTI processing, we used the *b*-value of 1,000 s/mm^2^ because the increased *b*-value leads to a decrease in signal-to-noise ratio (SNR) (41) that might affect the estimation of DTI metrics. For DKI processing, we used both the shells (*b* = 1,000 s/mm^2^ and b = 2,500 s/mm^2^ in addition to *b* = 0). As with all higher-order diffusion models, DKI requires multishell acquisition in order to increase the biological specificity of diffusion imaging (42, 43). The *b*-value optimisation for the DTI study is based on Soares et al. (15) and for the DKI study on Yan et al. (44).

Preprocessing of diffusion data was carried out using “topup” (45) and “eddy” (46) commands in FSL (FMRIB's Diffusion Toolbox). Susceptibility-induced distortions were first estimated using *topup*, based on pairs of reverse phase-encoded *b* = 0 images. The resulting susceptibility field was then incorporated into *eddy*, which performed simultaneous correction for eddy current–induced distortions and subject motion, while applying the previously estimated field to correct for susceptibility effects (47). Following these corrections, a brain mask was generated from the distortion-corrected diffusion data using the *bet2* command in FSL, with a fractional intensity threshold of 0.25 (48, 49). DTI metrics, FA, MD, AD and RD were obtained using dwi2tensor and tensor2metric commands in Mrtrix3 version 3.0.7 software (50). DKI metrics KFA, MK, AK and RK were obtained using the “tmi” command in Designer-v2 software (51). Each participant's FA map was then non-linearly registered to Montreal Neurological Institute (MNI) standard space using the tract-based spatial statistics (TBSS) toolkit of FSL (52). The FSL_HCP1065Fa 1 × 1 × 1 mm^3^ standard space image was used as a target. The rest of the DTI metrics (MD, AD, and RD) and DKI metrics (KFA, MK, AK, and RK) were normalized and warped by applying the deformations obtained from the FA co-registration.

### 2.5 Statistics

Voxel-based statistical analysis of FA, MD, AD, RD, KFA, MK, AK, and RK comparing ME/CFS, long COVID, and healthy controls was performed using the Statistical Parametric Mapping (SPM12) toolbox (53). Statistical inference was measured with the false discovery rate corrected cluster *p-value* (cluster *p-*FDR <0.05). Significant regions were overlaid on the T1-weighted image (mni_icbm152_t1_tal_nlin_sym_09a). Cluster locations were identified with the xjview toolbox (xjView | A viewing program for SPM) (54) and FSL software (55).

All data were assessed for normality using Shapiro-Wilk test. Only age was found to be normally distributed while all other clinical measures showed non-normal distribution (all *p* < 0.05). Age differences between ME/CFS, long COVID and healthy controls were analyzed using one-way ANOVA. Differences in clinical measures across three groups were examined using a generalized linear model. Multiple comparisons correction was adjusted using the Bonferroni correction method (56).

Correlation analysis between significant DTI and DKI metrics and clinical measures were performed using partial Spearman's rank correlation. All the statistical analyses were conducted using SPSS Statistics version 29.0, and *p*-values were adjusted for multiple comparisons using false discovery rate (57), with statistical significance defined as pFDR <0.05. Age and gender were included as covariates to control for their potential effects in all the analysis. Illness duration was included as an additional covariate when examining group differences between ME/CFS and long COVID.

DTI comparisons between long COVID participants and healthy controls were excluded to avoid redundancy and reduce the risk of type I error, as these data were already reported in a previous publication (34).

## 3 Results

### 3.1 Demographic and clinical characteristics

Group comparisons revealed significant differences across demographic and clinical measures (Table 1). Long COVID participants were older than controls (47.9 ± 13.3 vs. 37.9 ± 10.4 years, *p* = 0.021), and illness duration was significantly longer in ME/CFS than long COVID (14.5 ± 12.3 vs. 0.8 ± 0.7 years, ***p < 0.001***).

Across SF-36 domains, ME/CFS and long COVID patients scored significantly lower than controls on all subscales (general health, physical functioning, role physical, role emotional, pain, mental health, vitality and social functioning) (all *p* ≤ *0.05*) and ME/CFS showed significant impairment compared to long COVID on general health, physical functioning, role physical, vitality and social functioning (all *p* ≤ *0.05*).

Across WHODAS domains, ME/CFS and long COVID patients scored significantly lower than controls on all subscales (cognitive impairment, mobility, selfcare, interpersonal, life activity and society) (all *p* ≤ *0.01*), and ME/CFS showed significant impairment compared to long COVID on cognitive impairment, life activity and societal participation (all *p* ≤ *0.025*).

### 3.2 DTI group comparison

#### 3.2.1 ME/CFS vs. long COVID

No significant differences in the DTI metrics were observed between ME/CFS and long COVID.

#### 3.2.2 ME/CFS vs. healthy controls

**Figure 1:**
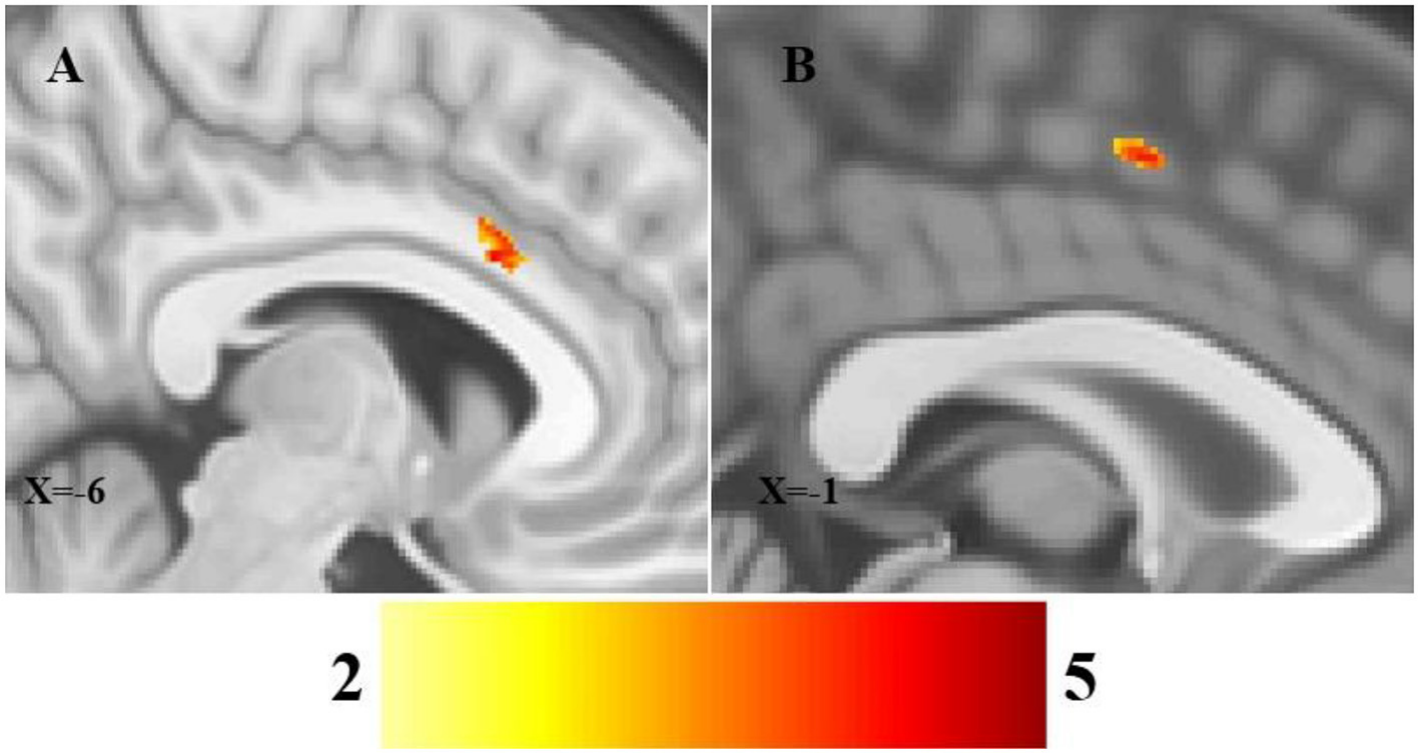
Findings of DTI in ME/CFS compared to healthy controls (HC). **(A)** significantly increased fractional anisotropy (FA) in the left cingulum (ME/CFS > HC) and **(B)** significantly decreased mean diffusivity, axial diffusivity and radial diffusivity in the left medial supplementary motor area/ medial frontal gyrus (ME/CFS <HC).

Voxel-based analysis of DTI metrics revealed (A) significantly increased FA (*p*-FDR = 0.024, Peak *T* = 4.42, Cluster size = 99, *X* = −6, *Y* = 16, and *Z* = 28; see Figure 1A, Table 2) in the left cingulum of ME/CFS compared to healthy controls, and (B) significantly decreased MD (*p*-FDR = 0.019, Cluster size = 109, Peak *T* = 4.26), AD (*p*-FDR = 0.034, Cluster size = 67, Peak *T* = 4.08) and RD (*p*-FDR = 0.032, Cluster size = 105, Peak *T* = 3.94) at the same location (*X* = −1, *Y* = 2, and *Z* = 50, see Figure 1B, Table 2) in the left supplementary motor area (SMA-L) of the medial frontal gyrus in ME/CFS compared to healthy controls.

**Table 2:** Significant DTI metrics cluster: ME/CFS vs. Healthy controls.

DTI Metric	Increased (↑)/ Decreased (↓)	Area	MNI coordinates x y z (mm)	Cluster size (voxels)	Peak *T*-value	Cluster *p*-FDR
**FA**	↑	Left cingulum	−6 16 28	99	4.42	0.024
**MD**	↓	SMA-L	−1 2 50	109	4.26	0.019
**AD**	↓	SMA-L	−1 2 50	67	4.08	0.034
**RD**	↓	SMA-L	−1 2 50	105	3.94	0.032

FA, represent Fractional Anisotropy; MD, Mean Diffusivity; AD, Axial Diffusivity; RD, Radial Diffusivity; Cingulum-L, Cingulum-Left; SMA-L, Supplementary motor area of left side, ↑: increased, ↓: decreased, FDR: False discovery rate.

### 3.3 DKI group comparison

#### 3.3.1 ME/CFS vs. long COVID

**Figure 2:**
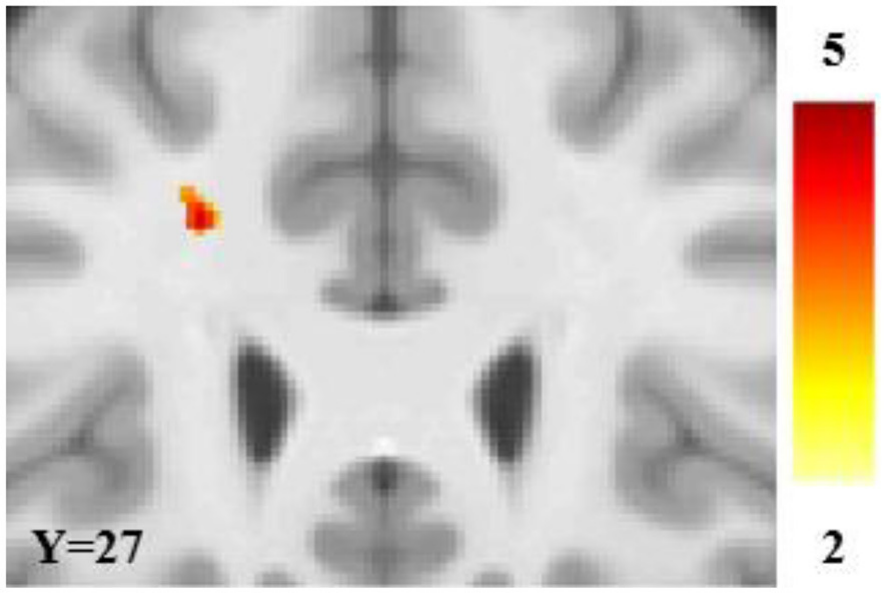
Comparison of ME/CFS and long COVID (ME/CFS <long COVID) showing significant increase in axial kurtosis (AK) in the left corona radiata.

AK was significantly increased in the left corona radiata of ME/CFS (*p*-FDR = 0.001, Cluster size = 107, Peak *T* = 4.65, *X* = −22, *Y* = 27 and *Z* = 26; *see* Figure 2) compared to long COVID.

#### 3.3.2 ME/CFS vs. healthy controls

**Figure 3:**
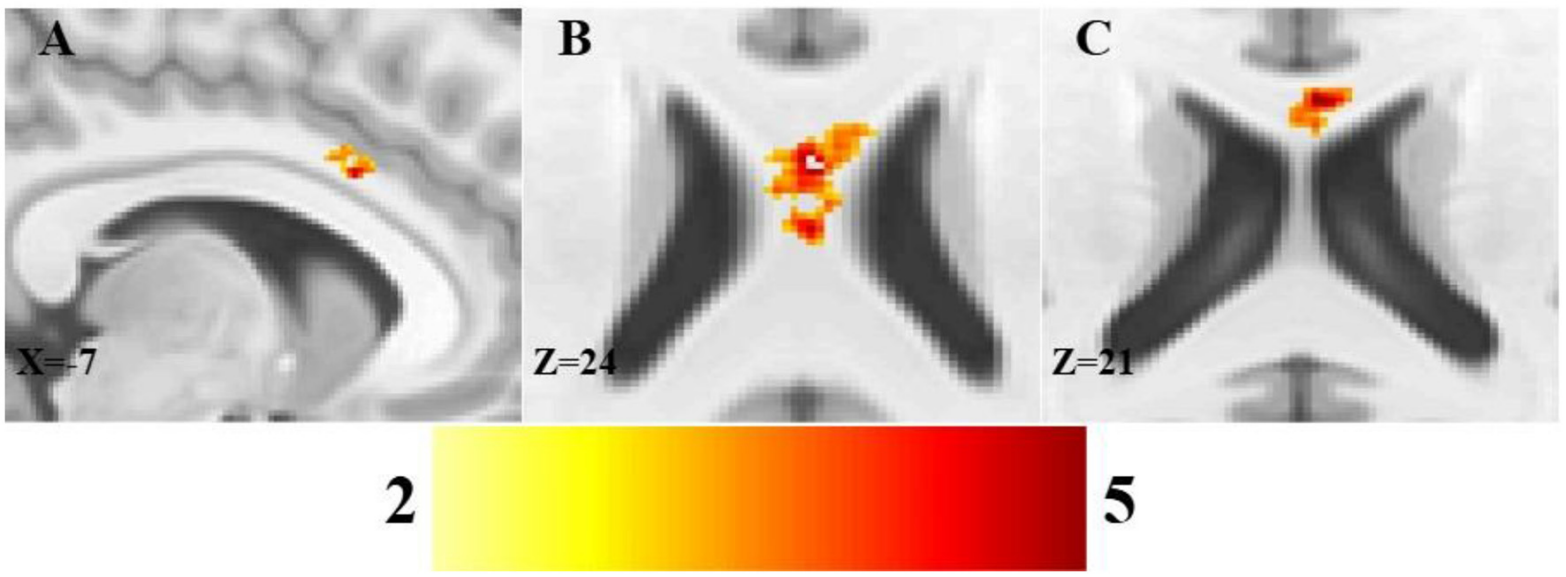
Findings of DKI in ME/CFS compared to healthy controls (HC) showing **(A)** significantly increased kurtosis fractional anisotropy in the left cingulum (ME/CFS > healthy controls) **(B)** significantly decreased axial kurtosis in the body of the corpus callosum (ME/CFS > healthy controls) and **(C)** significantly decreased axial kurtosis in the genu of the corpus callosum (ME/CFS <healthy controls).

Voxel-based analysis of DKI metrics revealed significantly increased KFA (*p*-FDR = 0.021, Peak *T* = 4.71, Cluster size = 98, *X* = −7, *Y* = 16, and *Z* = 29; see Figure 3A, Table 3) in the left cingulum of ME/CFS compared to healthy controls. Also, AK was significantly decreased in the body (*p*-FDR <0.001, Peak *T* = 5.42, Cluster size = 247, *X* = 1, *Y* = −3, and *Z* = 24; see Figure 3B, Table 3) and genu (*p*-FDR <0.001, Peak *T* = 5.02, Cluster size = 113, *X* = 2, *Y* = 12 and *Z* = 21; *see* Figure 3C, Table 3) of corpus callosum in ME/CFS compared to healthy controls. There were no significant differences in the MK and RK metrics.

**Table 3:** Significant DKI metrics cluster: ME/CFS vs. Healthy Controls.

DKI metric	Increased (↑)/ Decreased (↓)	Area	MNI coordinates x y z (mm)	Cluster size (voxels)	Peak *T*-value	Cluster *p*-FDR
**KFA**	↑	Left Cingulum	−7 16 29	98	4.71	0.021
**MK**	N/A	N/A	N/A	N/A	N/A	N/A
**AK**	↓	Genu of CC	2 12 21	113	5.02	0.000
	↓	Body of CC	1 −3 24	247	5.42	0.000
**RK**	N/A	N/A	N/A	N/A	N/A	N/A

KFA, Kurtosis Fractional Anisotropy; MK, Mean Kurtosis; AK, Axial Kurtosis; RK, Radial Kurtosis; CC, Corpus Callosum; ↑: increased, ↓: decreased, FDR: False discovery rate.

#### 3.3.3 Long COVID vs. healthy controls

**Figure 4:**
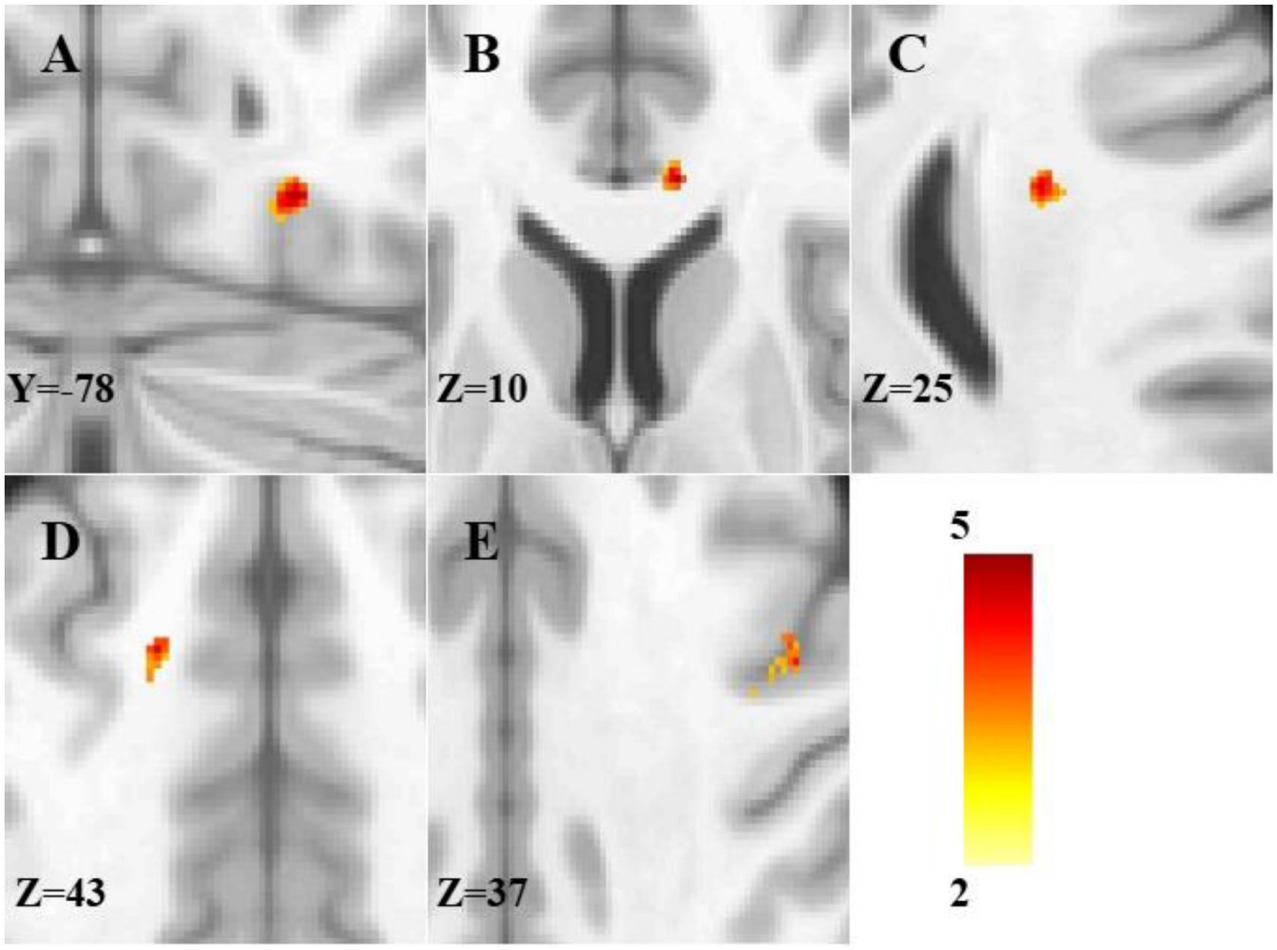
Long COVID compared to healthy controls (long COVID > healthy controls) showing **(A)** significant increase in mean kurtosis and radial kurtosis in right fusiform gyrus. **(B–E)** Significant increment in radial kurtosis in **(B)** Genu of corpus callosum/ Forceps minor, **(C)** Right superior corona radiata. **(D)** left superior corona radiata and **(E)** right precentral gyrus.

In DKI of long COVID compared to the healthy controls, we observed significantly increased MK (*p*-FDR = 0.024, Peak *T* = 4.93, Cluster size = 85, *X* = 27, *Y* = −78 and *Z* = −5; see Figure 4A, Table 4) and RK (*p*-FDR = 0.006, Peak *T* = 4.66 Cluster size = 87, *X* = 26, *Y* = −78 and Z = −5; see Figure 4A, Table 4) in the right fusiform gyrus. RK was increased in the genu of the corpus callosum (*p*-FDR = 0.001, Peak *T* = 4.76, Cluster size = 125, *X* = 10, *Y* = 32 and *Z* = 10; see Figure 4B, Table 4), right superior corona radiata (*p*-FDR = 0.006, Peak *T* = 4.61, Cluster size = 79, *X* = 28, *Y* = −1 and *Z* = 25; *see* Figure 4C, Table 4), left superior corona radiata (*p*-FDR = 0.012, Peak *T* = 4.53, Cluster size = 69, *X* = −15, *Y* = 25 and *Z* = 43; see Figure 4D, Table 4) and in right precentral gyrus (*p*-FDR = 0.006, Peak *T* = 4.28, Cluster size = 87, *X* = 48, *Y* = 2 and *Z* = 37; see Figure 4E, Table 4) in long COVID compared to healthy controls.

**Table 4:** Significant DKI metrics cluster: long COVID vs. Healthy controls.

DKI metric	Increased (↑)/ Decreased (↓)	Area	MNI coordinates x y z (mm)	Cluster size (voxels)	Peak *T*-value	Cluster *p*-FDR
**KFA**	N/A	N/A	N/A	N/A	N/A	N/A
**MK**	↑	Right fusiform gyrus	27 −78 −5	85	4.93	0.024
**AK**	N/A	N/A	N/A	N/A	N/A	N/A
**RK**	↑	Genu of CC/ forceps minor	10 32 10	125	4.76	0.001
	↑	Right superior corona radiata	28 −1 25	79	4.61	0.006
	↑	Left superior corona radiata	−15 25 43	69	4.54	0.012
	↑	Right precentral gyrus	48 2 37	66	4.28	0.013
	↑	Right fusiform gyrus	26 −78 −5	87	4.66	0.006

KFA, Kurtosis Fractional Anisotropy; MK, Mean Kurtosis; AK, Axial Kurtosis; RK, Radial Kurtosis; CC, Corpus Callosum; ↑: increased, ↓: decreased, FDR: False discovery rate, N/A: Not applicable.

### 3.4 Correlation analysis with clinical measures

We found significant uncorrected correlations between DTI, DKI metrics and clinical measures in ME/CFS and long COVID. However, we did not find any significant differences between these metrics after adjusting for multiple comparisons. All the correlations analysis between DTI, DKI and clinical measures are provided as a Supplementary Table 1–10.

## 4 Discussion

This is the first study to characterize brain tissue microstructural alterations in people living with ME/CFS and long COVID using both DTI and DKI methods. We identified distinct microstructural alterations between ME/CFS, long COVID and healthy controls. DKI offered valuable insights that complemented DTI findings.

### 4.1 Group Comparison: ME/CFS vs. Long COVID

We found significant differences in the DKI metric between ME/CFS and long COVID. There was a significant increase in AK in the left corona radiata, which is the major projection fiber and includes corticospinal, corticopontine and thalamocortical fibers (58). Yu et al. (59) in their DTI based study also reported higher AD in the major association fiber in ME/CFS. Elevated AK typically reflects greater diffusion complexity or restriction along the axonal axis, which is consistent with increased cellular packing, a more constrained microenvironment, or heightened structural complexity within axons (60). The microstructural alterations observed in the left corona radiata may therefore represent compensatory increases in cellular density or neuroinflammatory processes (61, 62). In addition, the corticospinal, corticobulbar, and thalamocortical pathways, which traverse the corona radiata are essential for voluntary motor control, fine and gross motor coordination, and sensory gating (63–65). Consequently, disruptions in the microstructure of the corona radiata could plausibly contribute to the motor difficulties, altered sensory processing, and cognitive impairment observed in these conditions.

Notably, our DTI analysis revealed no significant differences between ME/CFS and long COVID. This underscores the ability of DKI to overcome critical DTI limitations such as the inability to resolve crossing fibers (66) and, higher test-retest variability (67).

### 4.2 Group comparison: ME/CFS vs. healthy controls

Our DTI study found significantly decreased MD, AD and RD in the left SMA and increased FA in the left cingulum of ME/CFS compared to healthy controls. Our findings align with previous findings where decreased AD and MD and also increased FA were reported in people living with ME/CFS compared to healthy controls (28). Similarly, Wu et al. demonstrated microstructural changes in the left cingulum in ME/CFS (68). The SMA is located in the medial aspect of the superior frontal gyrus, involved in higher-order cognition, including planning, initiation and execution of complex voluntary actions and higher-order processes such as speech and motor sequencing (69). Similarly, the cingulum is a large association fiber pathway and also a part of the limbic system, which connects cingulate gyrus to the frontal, parietal, temporal and occipital cortices, allowing for integration of memory, emotion and executive function (70, 71). The decrease of MD, AD, and RD in the SMA could indicate an alteration in myelination and cellular density of the membrane, probably indicating cellular swelling or cellular proliferation (28, 72, 73). Furthermore, increased in FA in the cingulum could reflect increased myelination or cellular edema (74). Therefore, alterations in these metrics in the SMA and cingulum could cause cognitive deficits in ME/CFS.

The KFA parameter was also increased in the left cingulum of ME/CFS compared to healthy controls. This could be because KFA is a natural extension of the FA concept in the kurtosis tensor, which is mathematically analogous (75).

The AK parameter was significantly decreased in the genu and body of the corpus callosum in ME/CFS compared to healthy controls. The corpus callosum is the largest commissural white matter tract, connecting the two cerebral hemispheres (76–78). The genu of the corpus callosum primarily links the left and right prefrontal cortices involved in higher cognitive functions and decision making, while the body connects the frontal and parietal lobes, coordinating motor and sensory function (76–78). The decreased AK in the genu and body of the corpus callosum probably means there is axonal injury or loss of integrity causing alteration in axonal pathways (79). Therefore, ME/CFS presentation with cognitive dysfunction, motor slowing and hypersensitivity to noise and light (80, 81), is consistent with involvement of the corpus callosum.

### 4.3 Group comparison: long COVID vs. healthy controls

We found a significant increase in the RK values in the forceps minor and right and left superior corona radiata in long COVID compared to healthy controls. Forceps minor is an interhemispheric fiber tract that forms the genu of the corpus callosum and links both orbitofrontal cortices (82) while the right and left superior corona radiata are projection fibers that connect cortex to brainstem and thalamus with both afferent and efferent fibers (83). Involvement of commissural and projection fibers has been described in the previous post COVID-19 study (84). Two studies have implicated forceps minor in relation to cognitive dysfunction in the post COVID condition (85, 86). Additionally, a study also showed a relationship between the corona radiata, fatigue and cognitive complaints (87). Increased RK in commissural and projection fibers indicates enhanced restriction of water diffusion perpendicular to the principal fiber orientation, suggesting increased microstructural complexity in the radial direction. This pattern may arise from myelin compaction, reduced extracellular space, or glial-related alterations rather than axonal loss (88–90). As forceps minor and corona radiata are tracts central to cognition, attention, fatigue and emotional regulation (63–65, 86), impairment in these regions may contribute to the cognitive decline and fatigue in long COVID.

We also found increased RK in the right precentral gyrus and in the right fusiform gyrus. The precentral gyrus is the anatomical location of the primary motor cortex, which is responsible for controlling voluntary motor movement of the contralateral side of the body (91). Diez-Cirarda et al. (87) showed a relationship between reduction in the gray matter volume in the precentral gyrus and cognitive decline which is one of the hallmark symptoms of long COVID. Troll et al. (92) using fMRI found increased functional connectivity between the right caudate nucleus and both left and right precentral gyrus in long COVID compared to healthy controls. Additionally, increased T1/T2 signal intensity was reported in the precentral gyrus in long COVID compared to healthy controls, which is related to the higher myelin signal (34) consistent with our RK findings (88–90).

We observed significantly increased MK in the right fusiform gyrus, a region critical for visual processing and face recognition. Prior studies showed atrophy in the left fusiform gyrus (93), and right-sided involvement was associated with higher levels of COVID-related post-traumatic stress and social anxiety (94) and a prosopagnosia in long COVID (95). The elevated MK likely reflects greater tissue complexity, increased cellularity, or more restricted water diffusion, potentially due to neuroinflammation or reactive gliosis (62, 96). As no prior long COVID studies have utilized DKI, these findings represent a novel discovery of tissue microstructural alterations in people living with ME/CFS and long COVID.

## 5 Limitations

Despite novel findings in ME/CFS and long COVID, this study has some limitations. We did not stratify ME/CFS and healthy controls based on prior COVID infection. Additionally, as this is a cross-sectional study, future longitudinal research will be important to clarify whether the observed changes in DTI and DKI measures evolve or remain stable. Additionally, the long COVID group was relatively small (*n* = 19), which limits statistical power and the confidence of group comparisons. Therefore, these findings should be viewed as preliminary and will require confirmation in larger, well-powered samples. Furthermore, although age and sex were included as covariates, the significant age difference between groups may introduce residual confounding, particularly given the known sensitivity of diffusion metrics to age-related microstructural changes. Also, we were unable to formally compare the sensitivity of DTI and DKI, so differences between the methods may reflect acquisition or processing factors rather than true biological effects. DKI should therefore be viewed as providing complementary information, and dedicated studies are needed to assess comparative sensitivity. Additionally, we also excluded DTI comparisons between the long COVID and control groups due to prior publication (34), which may limit the direct comparisons across all groups.

## 6 Conclusions

This study identifies distinct tissue microstructural alterations in people living with ME/CFS and long COVID using DTI and DKI. These findings offer vital insights into the neuropathological basis of shared symptoms in both conditions. Additionally, our results suggest that DKI enables a more nuanced characterization of the complex neuropathological processes underlying these conditions. Complementary DTI and DKI insights contribute to a more comprehensive assessment of the associated microstructural changes.

## Data Availability

The raw data supporting the conclusions of this article will be made available by the authors, without undue reservation.
