# Supplementary material for: Microstructural alterations in brain tissue of ME/CFS and long COVID using diffusion tensor imaging and diffusion kurtosis imaging
Source: Front Med (Lausanne). 2026 Jul 17;13:1824498. doi: 10.3389/fmed.2026.1824498 (PMC13424405; doi:10.3389/fmed.2026.1824498)
Supplement: Supplementary file 1 [file Table_1.DOCX]

| **Table 1: Correlation analysis between DTI and DKI parameters and Clinical Measures in ME/CFS (with FDR correction).** DTI: Diffusion Tensor Imaging, DKI: Diffusion Kurtosis Imaging, FA: Fractional Anisotropy, AK: Axial Kurtosis, RK: Radial Kurtosis, SF 36 : 36 item short form health survey, WHODAS: World Health Organization Disability Assessment Schedule, FDR: False Discovery Rate. | | | | | | |
| --- | --- | --- | --- | --- | --- | --- |
| Clinical Measures | | DTI/DKI METRIC | Brain regions | Uncorrected P-values | pFDR | r value |
| SF36 | Physical functioning | FA | Cingulum-L | 0.038 | 0.618 | 0.492 |
|  | Vitality | AK | Body of corpus callosum | 0.024 | 0.618 | 0.528 |
|  |  |  |  |  |  |  |
| WHODAS | Mobility | AK | Left corona radiata | 0.033 | 0.248 | 0.466 |
| DURATION OF ILLNESS | | | | NOT SIGNIFICANT | | |
| FATIGUE SEVERITY | | AK | Genu of corpus callosum | 0.007 | 0.112 | 0.315 |
|  | | AK | Left corona radiata | 0.033 | 0.248 | 0.446 |

| **Table 2: Correlation analysis between DKI parameters and Clinical Measures in long COVID (with FDR correction).** DKI: Diffusion Kurtosis Imaging, MK: Mean Kurtosis, AK: Axial Kurtosis, RK: Radial Kurtosis, SF 36 : 36 item short form health survey, WHODAS: World Health Organization Disability Assessment Schedule, FDR: False Discovery Rate. | | | | | | |
| --- | --- | --- | --- | --- | --- | --- |
| Clinical Measures | | DKI PARAMETERS | Brain regions | Uncorrected P-values | pFDR | r value |
| SF36 | General Health | RK | Left superior corona radiata | 0.006 | 0.168 | -0.795 |
|  | General Health | RK | Forceps Minor | 0.002 | 0.096 | -0.843 |
|  | Vitality | RK | Precentral Gyrus | 0.037 | 0.280 | 0.662 |
|  | Mental Health | AK | Forceps minor | 0.018 | 0.266 | -0.722 |
|  | Social functioning | AK | Forceps minor | 0.007 | 0.168 | -0.784 |
|  | Vitality | RK | Precentral gyrus | 0.025 | 0.266 | -0.69 |
|  | Role Emotional | RK | Precentral gyrus | 0.038 | 0.28 | -0.65 |
|  | Social Functioning | RK | Precentral gyrus | 0.001 | 0.096 | -0.86 |
| WHODAS | Mobility | RK | Superior corona radiata - R | 0.029 | 0.243 | -0.76 |
|  | Mobility | RK | Forceps Minor | 0.011 | 0.143 | -0.83 |
|  | Interpersonal | RK | Superior longitudinal fasciculus - L | 0.039 | 0.244 | -0.77 |
|  | Society | RK | Superior longitudinal fasciculus - L | 0.031 | 0.243 | -0.67 |
| DURATION OF ILLNESS |  | AK | Superior Longitudinal fasciculus | 0.048 | 0.608 | -0.48 |
| FATIGUE SEVERITY | | | | NOT SIGNIFICANT | | |

| **Table 3: Correlation analysis with all uncorrected p values: ME/CFS vs SF36.** DTI: Diffusion Tensor Imaging, DKI: Diffusion Kurtosis Imaging, FA: Fractional Anisotropy, KFA: Kurtosis Fractional Anisotropy, AD: Axial Diffusivity, MD: Mean Diffusivity, RD: Radial Diffusivity, AK: Axial Kurtosis,MK: Mean Kurtosis, RK: Radial Kurtosis,SMA: Supplementory Motor Area, SF 36 : 36 item short form health survey, FDR: False Discovery Rate. | | | | | | | | | | |
| --- | --- | --- | --- | --- | --- | --- | --- | --- | --- | --- |
| Brain Regions | DTI/DKI Parameter |  | General_Health | Physical_functioning | Role_physical | Role_emotional | Pain | Mental_health | Vitality | Social_functioning |
| Cingulum | FA | r | 0.177 | 0.492 | 0.042 | 0.112 | -0.075 | 0.190 | 0.010 | 0.430 |
|  |  | P values | 0.483 | 0.038 | 0.869 | 0.659 | 0.767 | 0.450 | 0.969 | 0.085 |
|  |  | df | 16 | 16 | 16 | 16 | 16 | 16 | 16 | 15 |
| Cingulum | KFA | r | 0.120 | 0.430 | 0.036 | 0.090 | -0.018 | 0.141 | -0.024 | 0.423 |
|  |  | P values | 0.635 | 0.075 | 0.886 | 0.723 | 0.944 | 0.578 | 0.924 | 0.090 |
|  |  | df | 16 | 16 | 16 | 16 | 16 | 16 | 16 | 15 |
| SMA | AD | r | -0.094 | 0.098 | 0.197 | -0.068 | 0.099 | -0.294 | -0.311 | 0.054 |
|  |  | P values | 0.712 | 0.698 | 0.433 | 0.789 | 0.695 | 0.237 | 0.210 | 0.838 |
|  |  | df | 16 | 16 | 16 | 16 | 16 | 16 | 16 | 15 |
| SMA | MD | r | -0.034 | 0.076 | 0.165 | -0.040 | 0.099 | -0.316 | -0.293 | 0.034 |
|  |  | P values | 0.894 | 0.763 | 0.514 | 0.876 | 0.695 | 0.201 | 0.238 | 0.897 |
|  |  | df | 16 | 16 | 16 | 16 | 16 | 16 | 16 | 15 |
| SMA | RD | r | -0.128 | 0.044 | 0.187 | 0.031 | 0.117 | -0.254 | -0.370 | 0.050 |
|  |  | P values | 0.613 | 0.862 | 0.458 | 0.902 | 0.644 | 0.308 | 0.131 | 0.848 |
|  |  | df | 16 | 16 | 16 | 16 | 16 | 16 | 16 | 15 |
| Corpus_callosum_body | AK | r | 0.448 | 0.375 | 0.321 | 0.058 | -0.053 | 0.223 | 0.528 | 0.279 |
|  |  | P values | 0.062 | 0.126 | 0.194 | 0.818 | 0.834 | 0.375 | 0.024 | 0.278 |
|  |  | df | 16 | 16 | 16 | 16 | 16 | 16 | 16 | 15 |
| Corpus_callosum_Genu | AK | r | 0.420 | 0.265 | 0.186 | 0.224 | 0.365 | 0.054 | 0.302 | 0.000 |
|  |  | P values | 0.083 | 0.288 | 0.460 | 0.371 | 0.136 | 0.831 | 0.223 | 0.999 |
|  |  | df | 16 | 16 | 16 | 16 | 16 | 16 | 16 | 15 |
| Left Corona Radiata | AK | r | 0.284 | -0.220 | -0.021 | 0.276 | 0.423 | -0.045 | -0.015 | 0.059 |
|  |  | P values | 0.212 | 0.339 | 0.929 | 0.227 | 0.056 | 0.846 | 0.947 | 0.811 |
|  |  | df | 19 | 19 | 19 | 19 | 19 | 19 | 19 | 17 |

| **Table 4: Correlation analysis with all uncorrected p values: ME/CFS vs WHODAS.** DTI: Diffusion Tensor Imaging, DKI: Diffusion Kurtosis Imaging, FA: Fractional Anisotropy, KFA: Kurtosis Fractional Anisotropy, AD: Axial Diffusivity, MD: Mean Diffusivity, RD: Radial Diffusivity, AK: Axial Kurtosis,MK: Mean Kurtosis, RK: Radial Kurtosis,SMA: Supplementory Motor Area, WHODAS: World Health Organization Disability Assessment Schedule. | | | | | | | | |
| --- | --- | --- | --- | --- | --- | --- | --- | --- |
| Brain Regions | DTI/DKI Parameters | **WHODAS DOMAINS** | **Cognitive** | **Mobility** | **Selfcare** | **interpersonal** | **lifeactivity** | **society** |
| Cingulum | FA | r | -0.008 | 0.184 | 0.275 | 0.409 | 0.158 | 0.037 |
|  |  | P values | 0.970 | 0.413 | 0.216 | 0.058 | 0.483 | 0.870 |
|  |  | df | 20 | 20 | 20 | 20 | 20 | 20 |
| Cingulum | KFA | r | -0.093 | 0.129 | 0.241 | 0.345 | 0.129 | -0.002 |
|  |  | P values | 0.681 | 0.568 | 0.280 | 0.116 | 0.567 | 0.993 |
|  |  | df | 20 | 20 | 20 | 20 | 20 | 20 |
| SMA | AD | r | 0.090 | 0.219 | 0.134 | -0.023 | 0.122 | 0.030 |
|  |  | P values | 0.689 | 0.327 | 0.552 | 0.920 | 0.589 | 0.894 |
|  |  | df | 20 | 20 | 20 | 20 | 20 | 20 |
| SMA | MD | r | 0.077 | 0.257 | 0.165 | 0.001 | 0.132 | 0.042 |
|  |  | P values | 0.733 | 0.247 | 0.463 | 0.997 | 0.559 | 0.852 |
|  |  | df | 20 | 20 | 20 | 20 | 20 | 20 |
| SMA | RD | r | 0.023 | 0.217 | 0.126 | -0.024 | 0.078 | 0.009 |
|  |  | P values | 0.919 | 0.331 | 0.577 | 0.916 | 0.731 | 0.967 |
|  |  | df | 20 | 20 | 20 | 20 | 20 | 20 |
| Body of corpus callosum | AK | r | -0.199 | -0.048 | -0.165 | -0.060 | -0.251 | -0.222 |
|  |  | P values | 0.374 | 0.831 | 0.462 | 0.789 | 0.260 | 0.322 |
|  |  | df | 20 | 20 | 20 | 20 | 20 | 20 |
| Genu of corpus callosum | AK | r | -0.172 | -0.036 | -0.228 | 0.010 | -0.155 | 0.024 |
|  |  | P values | 0.443 | 0.875 | 0.308 | 0.964 | 0.491 | 0.914 |
|  |  | df | 20 | 20 | 20 | 20 | 20 | 20 |
| Left corona radiata | AK | r | 0.333 | 0.466 | -0.053 | 0.320 | 0.099 | 0.329 |
|  |  | P values | 0.152 | 0.033 | 0.857 | 0.157 | 0.725 | 0.145 |
|  |  | df | 18 | 19 | 12 | 19 | 13 | 19 |

| **Table 5: Correlation analysis with all uncorrected p values: Long COVID vs SF36.** DKI: Diffusion Kurtosis Imaging, AK: Axial Kurtosis,MK: Mean Kurtosis, RK: Radial Kurtosis, AK: Axial Kurtosis, FDR: False Discovery Rate. SF 36 : 36 item short form health survey, FDR: False Discovery Rate. | | | | | | | | | | |
| --- | --- | --- | --- | --- | --- | --- | --- | --- | --- | --- |
| **Brain Regions** | DKI parameters | **SF36 DOMAINS** | **Vitality** | **GeneralHealth** | **PhysicalFunctioning** | **RolePhysical** | **RoleEmotional** | **Pain** | **MentalHealth** | **SocialFunctioning** |
| Superior frontal white matter | MK | r | -0.475 | -0.347 | -0.520 | -0.645 | -0.267 | 0.058 | -0.179 | -0.523 |
|  |  | P values | 0.166 | 0.326 | 0.101 | 0.044 | 0.456 | 0.865 | 0.620 | 0.121 |
|  |  | df | 8 | 8 | 9 | 8 | 8 | 9 | 8 | 8 |
| Pons | MK | r | -0.094 | -0.043 | -0.016 | -0.090 | -0.403 | -0.134 | -0.166 | -0.081 |
|  |  | P values | 0.795 | 0.907 | 0.963 | 0.806 | 0.249 | 0.695 | 0.646 | 0.825 |
|  |  | df | 8 | 8 | 9 | 8 | 8 | 9 | 8 | 8 |
| Pons | MK | r | -0.211 | -0.486 | 0.073 | 0.195 | 0.585 | 0.429 | -0.100 | 0.215 |
|  |  | P values | 0.559 | 0.155 | 0.831 | 0.589 | 0.076 | 0.187 | 0.784 | 0.551 |
|  |  | df | 8 | 8 | 9 | 8 | 8 | 9 | 8 | 8 |
| Genu of corpus callosum | AK | r | -0.612 | 0.359 | -0.551 | -0.189 | -0.366 | -0.269 | -0.722 | -0.784 |
|  |  | P values | 0.060 | 0.308 | 0.079 | 0.601 | 0.298 | 0.424 | 0.018 | 0.007 |
|  |  | df | 8 | 8 | 9 | 8 | 8 | 9 | 8 | 8 |
| Inferior longitudinal fasciculus | AK | r | -0.282 | -0.024 | -0.676 | -0.161 | -0.081 | 0.128 | 0.362 | -0.197 |
|  |  | P values | 0.431 | 0.948 | 0.022 | 0.656 | 0.824 | 0.707 | 0.304 | 0.586 |
|  |  | df | 8 | 8 | 9 | 8 | 8 | 9 | 8 | 8 |
| Corona radiata |  | r | 0.133 | -0.704 | 0.142 | 0.230 | 0.616 | 0.303 | 0.353 | 0.595 |
|  |  | P values | 0.714 | 0.023 | 0.678 | 0.524 | 0.058 | 0.366 | 0.317 | 0.070 |
|  |  | df | 8 | 8 | 9 | 8 | 8 | 9 | 8 | 8 |
| Corona radiata | RK | r | -0.329 | -0.795 | 0.023 | 0.429 | 0.046 | -0.295 | -0.254 | 0.103 |
|  |  | P values | 0.353 | 0.006 | 0.947 | 0.216 | 0.900 | 0.379 | 0.479 | 0.778 |
|  |  | df | 8 | 8 | 9 | 8 | 8 | 9 | 8 | 8 |
| ForcepsMinor | RK | r | -0.347 | -0.843 | 0.041 | -0.193 | -0.128 | 0.006 | -0.343 | 0.002 |
|  |  | P values | 0.326 | 0.002 | 0.906 | 0.593 | 0.725 | 0.986 | 0.331 | 0.996 |
|  |  | df | 8 | 8 | 9 | 8 | 8 | 9 | 8 | 8 |
| Precentral gyrus | RK | r | -0.698 | 0.009 | -0.545 | -0.564 | -0.659 | 0.063 | -0.196 | -0.863 |
|  |  | P values | 0.025 | 0.980 | 0.083 | 0.089 | 0.038 | 0.854 | 0.587 | 0.001 |
|  |  | df | 8 | 8 | 9 | 8 | 8 | 9 | 8 | 8 |
| Left corona radiata | AK |  | -0.664 | 0.200 | -0.055 | -0.347 | 0.081 | -0.647 | -0.379 | -0.120 |
|  |  |  | 0.051 | 0.605 | 0.888 | 0.360 | 0.824 | 0.060 | 0.315 | 0.759 |
|  |  |  | 7 | 7 | 7 | 7 | 8 | 7 | 7 | 7 |

| **Table 6: Correlation analysis with all uncorrected p values: Long COVID vs WHODAS.** DKI: Diffusion Kurtosis Imaging, AK: Axial Kurtosis,MK: Mean Kurtosis, RK: Radial Kurtosis, FDR: False Discovery Rate. WHODAS: World Health Organization Disability Assessment Schedule. | | | | | | | | |
| --- | --- | --- | --- | --- | --- | --- | --- | --- |
| **Brain regions** | DKI Parameters | **WHODAS** | **Mobility** | **LifeActivities** | **Interpersonal** | **Cognitive** | **Society** | **SelfCare** |
| Genu of corpus callosum | AK | r | 0.064 | 0.032 | 0.351 | 0.195 | 0.442 | 0.179 |
|  |  | P values | 0.880 | 0.940 | 0.440 | 0.566 | 0.201 | 0.578 |
|  |  | df | 6 | 6 | 5 | 9 | 8 | 10 |
| Corona radiata | RK | r | -0.596 | -0.019 | -0.530 | 0.006 | -0.161 | 0.031 |
|  |  | P values | 0.119 | 0.964 | 0.221 | 0.986 | 0.657 | 0.925 |
|  |  | df | 6 | 6 | 5 | 9 | 8 | 10 |
| ForcepsMinor | RK | r | -0.830 | -0.111 | -0.206 | -0.130 | -0.372 | -0.183 |
|  |  | P values | 0.011 | 0.793 | 0.657 | 0.702 | 0.289 | 0.570 |
|  |  | df | 6 | 6 | 5 | 9 | 8 | 10 |
| Left corona radiata | AK | r | -0.683 | -0.293 | -0.311 | -0.732 | -0.571 | -0.603 |
|  |  | P values | 0.062 | 0.632 | 0.453 | 0.160 | 0.139 | 0.113 |
|  |  | df | 6 | 3 | 6 | 3 | 6 | 6 |

| **Table 7: Correlation analysis with all uncorrected p values: ME/CFS vs Fatigue severity.** DTI: Diffusion Tensor Imaging, DKI: Diffusion Kurtosis Imaging, FA: Fractional Anisotropy, KFA: Kurtosis Fractional Anisotropy, AD: Axial Diffusivity, MD: Mean Diffusivity, RD: Radial Diffusivity, AK: Axial Kurtosis,MK: Mean Kurtosis, RK: Radial Kurtosis,SMA: Supplementory Motor Area, SF 36 : 36 item short form health survey, FDR: False Discovery Rate. | | | | | | | | | | |
| --- | --- | --- | --- | --- | --- | --- | --- | --- | --- | --- |
| **Brain regions** | | SMA | SMA | SMA | Precentral gyrus | Cingulum | Cingulum | Body of corpus callosum | Genu of corpus callosum | Left corona radiata |
| DTI/DKI parameters | | AD | MD | RD | RK | FA | KFA | AK | AK | AK |
| **Fatigue Severity** | r | -0.039 | -0.071 | -0.047 | -0.161 | -0.011 | 0.009 | -0.089 | -0.066 | 0.446 |
|  | P values | 0.867 | 0.761 | 0.840 | 0.486 | 0.962 | 0.970 | 0.702 | 0.777 | 0.033 |
|  | df | 19 | 19 | 19 | 19 | 19 | 19 | 19 | 19 | 21 |

| \| **Table 8: Correlation analysis with all uncorrected p values: Long COVID vs Fatigue severity.** DKI: Diffusion Kurtosis Imaging, AK: Axial Kurtosis,MK: Mean Kurtosis, RK: Radial Kurtosis, FDR: False Discovery Rate. \| \| --- \| | | | | | | | |
| --- | --- | --- | --- | --- | --- | --- | --- | --- |
| **Brain regions** | | Precentral gyrus | Forceps minor | Corona radiata | Superior frontal white matter | Left corona radiata | Genu of corpus callosum |
| DTI/DKI parameters | | RK | MK | RK | MK | AK | AK |
| **Fatigue Severity** | r | -0.047 | -0.161 | -0.077 | -0.066 | 0.050 | -0.241 |
|  | P values | 0.840 | 0.486 | 0.741 | 0.777 | 0.892 | 0.293 |
|  | df | 19 | 19 | 19 | 19 | 8 | 19 |

| **Table 9: Correlation analysis with all uncorrected p values: ME/CFS vs Duration of illness.** DTI: Diffusion Tensor Imaging, DKI: Diffusion Kurtosis Imaging, FA: Fractional Anisotropy, KFA: Kurtosis Fractional Anisotropy, AD: Axial Diffusivity, MD: Mean Diffusivity, RD: Radial Diffusivity, AK: Axial Kurtosis,MK: Mean Kurtosis, RK: Radial Kurtosis,SMA: Supplementory Motor Area, SF 36 : 36 item short form health survey, FDR: False Discovery Rate. | | | | | | | | |
| --- | --- | --- | --- | --- | --- | --- | --- | --- |
| **Brain regions** | | SMA | SMA | SMA | Cingulum | Cingulum | Body of corpus callosum | Genu of corpus callosum |
| DTI/DKI parameters | | AD | MD | RD | FA | KFA | AK | AK |
| **Fatigue Severity** | r | -0.034 | -0.115 | -0.131 | 0.050 | -0.013 | 0.103 | 0.017 |
|  | P values | 0.863 | 0.552 | 0.498 | 0.796 | 0.948 | 0.595 | 0.928 |
|  | df | 27 | 27 | 27 | 27 | 27 | 27 | 27 |

| \| **Table 10: Correlation analysis with all uncorrected p values: Long COVID vs Duration of illness.** DKI: Diffusion Kurtosis Imaging, AK: Axial Kurtosis,MK: Mean Kurtosis, RK: Radial Kurtosis, FDR: False Discovery Rate. \| \| --- \| | | | | | | | |
| --- | --- | --- | --- | --- | --- | --- | --- | --- |
| **Brain regions** | | Superior corona radiata | Forceps minor | Corona radiata | Superior frontal white matter | Left corona radiata | Genu of corpus callosum |
| DTI/DKI parameters | | RK | MK | RK | MK | AK | AK |
| **Duration of illness** | r | 0.246 | -0.161 | -0.077 | -0.066 | 0.050 | -0.241 |
|  | P values | 0.341 | 0.486 | 0.741 | 0.777 | 0.892 | 0.293 |
|  | df | 15 | 19 | 19 | 19 | 8 | 19 |
